# A Localization-Free Interference and Energy Holes Minimization Routing for Underwater Wireless Sensor Networks

**DOI:** 10.3390/s18010165

**Published:** 2018-01-09

**Authors:** Anwar Khan, Ismail Ahmedy, Mohammad Hossein Anisi, Nadeem Javaid, Ihsan Ali, Nawsher Khan, Mohammed Alsaqer, Hasan Mahmood

**Affiliations:** 1 Department of Electronics, Quaid-i-Azam University, Islamabad 44000, Pakistan; hasan@qau.edu.pk; 2 Department of Electronics, University of Peshawar, Peshawar KPK 25000, Pakistan; 3 Department of Computer System and Technology, Faculty of Computer Science and Information Technology, University of Malaya, Kualalumpur 50603, Malaysia; ihsanalichd@gmail.com; 4 School of Computer Science and Electronic Engineering, University of Essex, Colchester CO4 3SQ, UK; m.anisi@essex.ac.uk; 5 COMSATS Institute of Information Technology, Park Road, Islamabad 44000, Pakistan; nadeemjavaidqau@gmail.com; 6 Department of Computer Science, Abdul Wali Khan University, Mardan 23200, Pakistan; nawsherkhan@gmail.com; 7 Collage of Computer Science, King Khalid University, Abha 61421, Saudi Arabia; msalsaqr@kku.edu.sa

**Keywords:** underwater, routing, protocol, interference, energy holes, adaptive transmission range

## Abstract

Interference and energy holes formation in underwater wireless sensor networks (UWSNs) threaten the reliable delivery of data packets from a source to a destination. Interference also causes inefficient utilization of the limited battery power of the sensor nodes in that more power is consumed in the retransmission of the lost packets. Energy holes are dead nodes close to the surface of water, and their early death interrupts data delivery even when the network has live nodes. This paper proposes a localization-free interference and energy holes minimization (LF-IEHM) routing protocol for UWSNs. The proposed algorithm overcomes interference during data packet forwarding by defining a unique packet holding time for every sensor node. The energy holes formation is mitigated by a variable transmission range of the sensor nodes. As compared to the conventional routing protocols, the proposed protocol does not require the localization information of the sensor nodes, which is cumbersome and difficult to obtain, as nodes change their positions with water currents. Simulation results show superior performance of the proposed scheme in terms of packets received at the final destination and end-to-end delay.

## 1. Introduction

Overcoming interference and energy holes in underwater wireless sensor networks (UWSNs) usually guarantees reliable data transfer from a source to a destination. However, addressing these issues is linked with addressing the inherent challenges of underwater communications: low available bandwidth, greater propagation delay than terrestrial radio frequency communications and limited battery power [1]. However, the performance metrics of these networks are the same as for the terrestrial networks [2,3,4,5]. These networks find applications in offshore exploration, leak detection, seismic and equipment monitoring [6], military surveillance, underwater navigation, disaster prevention and environmental monitoring [7].

Underwater routing protocols that involve the mitigation of interference and energy holes are unique for a number of reasons. Interference results in packet collision that, in consequence, leads to packet loss. In a similar fashion, the formation of energy holes disconnects the routing traffic from a source to a destination, which also results in data loss. Such losses are unbearable in underwater communications, where sensor nodes already operate on limited battery power. Therefore, protocols coping with these issues provide the reliable delivery of data from a source to a destination. Such data delivery is particularly important in time-sensitive and military applications [8]. Specifically, when data loss due to interference is overcome, the limited battery power of nodes is also utilized in an effective and efficient fashion. The power that is lost against interference is then utilized to deliver more data packets. Likewise, when a forwarder node between a sender and a receiver dies and becomes an energy hole, it leads to a loss of data from the sender to the receiver. The data loss causes unnecessary power consumption of the sender node. Therefore, overcoming this energy hole ensures reliable data delivery as well as efficient power utilization.

The conventional routing protocols that cope with energy holes require that the localization information of an energy hole is known [9,10]. However, localization is a cumbersome and challenging task, as nodes change their positions with water currents. This leads to inaccuracy in the measurement of the position of an energy hole. Furthermore, an energy hole may change its position, and nodes may not detect it early because of the long delay in underwater communications. As a result, false position detection of all the energy holes may compromise the performance of the network. The protocols addressing the interference select routing paths that involve the least number of neighbors of a forwarder node [11,12]. However, with the least number of neighbors, a forwarder may not forward packets further when its neighbors die. In other words, the death of the least number of neighbors of a forwarder node results in the formation of energy holes. This also results in overall degradation of the network performance; in particular, the number of packets that reach the final destination decreases significantly. This is contrary to the routing protocols in which forwarders do not select the routing paths on the basis of the least number of neighbors and, therefore, have higher interference.

There are a number of challenges associated with the design of interference and energy holes minimization routing protocols. The underwater medium carries unpredictable and severe conditions that challenge underwater communications. These include noise, the mobility of sensor nodes with water, interference from underwater objects, shadow zones and attenuation of the desired signal  [13,14]. Specifically, the movement of nodes with water currents challenges the communications among nodes. This is because it becomes difficult to locate the positions of nodes when they are not stationary [15]. This becomes critically important for circumstances in which nodes die and live nodes have to replace their positions. This work addresses some of these challenges.

In this paper, the localization-free interference and energy holes minimization (LF-IEHM) protocol is proposed for UWSNs. The protocol selects forwarder nodes on the basis of the level of the water pressure. Nodes close to the water surface have low water pressure and are preferred for selection as forwarders. If two or more more expected forwarder nodes have the same pressure levels, the response time is taken into account to choose the best forwarder. The response time is a measure of the distance of a forwarder node from the source node. This strategy reduces the end-to-end delay and ensures that packets follow the shortest routes from a source to a destination. The proposed protocol uses a variable transmission range of sensor nodes. A node can increase its transmission range to include one or more live forwarder nodes for a situation in which it does not find any node within its transmission range. This controls the energy hole problem and reduces packet loss. This strategy is particularly effective under sparse conditions. Additionally, the proposed protocol does not require the position information of energy holes to be known, and it adjusts the holding time in a manner that minimizes simultaneous packets’ transmission by more than one node. This reduces interference, which, in turn, further reduces packet loss.

The proposed protocol contributes in two ways. Firstly, it minimizes the existence of energy holes (conditions in which sender nodes do not find any neighbor node for data forwarding or when all the neighbors are dead). A sender node with no neighbor increases its transmission range to include one or more neighbors for data forwarding. This strategy establishes a path from the bottom to the surface of water during data forwarding that, in turn, avoids packet loss. At the same time, it does not require the full-dimensional position information of the sensor nodes that the conventional routing protocols take into account. Secondly, to avoid interference during packet forwarding, a unique packet holding time is defined for every node to reduce the probability of two or more nodes forwarding packets at the same time. This ensures reduction in the loss of packets due to interference during data forwarding.

## 2. Related Work and Our Contribution

The authors in [16] propose a novel energy efficient protocol (NEFP) that selects forwarder nodes within a restricted zone from a source to a destination. Nodes that are close to the destination are given preference to take part in the routing process. The forwarding probability of a packet is also calculated along the expected routing path using Markov chains. The time for which a node holds a received packet is set so as to reduce interference. However, it requires the localization information of sensor nodes, which consumes surplus energy to locate the positions of nodes. Additionally, localization is difficult to achieve in underwater communications, as nodes change their positions frequently with water currents, and nodes within the restricted forwarding zone die early because of frequent selection as forwarders. In order to avoid interference and reduce void hole formation, a routing protocol is proposed in [17]. The protocol selects forwarder nodes on the basis of a cost function. The cost function is calculated for every node on the basis of its number of neighbors, the hop count and its distance form the sender. A sender node selects a forwarder node among its neighbors having the greatest value of the cost function. However, the protocol requires localization information of the sensor nodes. The energy hole repairing-depth-based routing (EHRDBR) [18] considers the death of low-depth nodes in the depth-based routing (DBR) protocol [19]. In DBR, the low-depth nodes die early because of frequent selection as forwarders. This creates energy holes in the network, which affects the delivery of the packets to the sink. EHRDBR detects an energy hole and replaces it with a live node. This avoids the loss of data packets. However, the detection of an energy hole and the moving of a live node to its exact position are cumbersome. These require localization information, which is challenging to acquire in underwater communications. The authors in [20] propose a routing protocol based on the Markov model to mitigate the channel noise and interference in underwater communications. The probability of a path is first found on the basis of its stability, its adaptation with respect to the changing traffic of data and the number of hops (less hops are preferred). The data is then routed along the best selected path. However, as a result of nodes’ movements and the unreliability of the links in underwater channels, the properties of a selected path may change within the time of calculation of the path and its selection as a route. This, in consequence, results in the loss of the effectiveness of the selected path for data transmission. The opportunistic void avoidance routing (OVAR) protocol forwards packets from a source to a destination using opportunistic routing [21]. The relay nodes are selected on the basis of the successful delivery probability of a packet and packet advancement. However, the protocol involves the localization information of sensor nodes. It also suffers from the early death of nodes close to the water surface.

A channel-aware routing protocol is proposed in [22] to avoid the zones in which forwarders are not available and in which the shadowing effect prevails (shadow zones). The protocol combines the history of nodes with successful packets’ transmission, the number of hops and the power control to select the candidate forwarder nodes. The proposed protocol achieves high throughput and energy efficiency. However, its performance is compromised in dense networks in terms of high end-to-end delay. This is due to the constant checking of successful packets’ transmission history. The hydrocast protocol proposed in [23] uses depth-based opportunistic routing along with a dead and recovery method.The protocol aims to mitigate the interference and energy consumption in the underwater data routing of nodes. The protocol uses a recovery method to route data packets from a node that is within the void zone; a node has no neighbors at all that have lower pressure levels than the node itself. In this case, a recovery path is established from the node in the void zone through the route-discovery method. In the route-discovery method, a node in the void zone forwards data packets to another node positioned to its side. The forwarder node then either greedily forwards the packet further or forwards it in the same manner to another sidewise neighbor. This process carries on until the packet reaches the sink. However, the protocol suffers from an early death of nodes due to opportunistic routing. The interference-aware inverse energy-efficient depth-based routing (IA-IEEDBR) protocol addresses the minimization of interference in underwater routing [24]. The protocol routes data by selecting forwarder nodes having the lowest residual energy, the least number of neighbors and the least depth. However, the selection of nodes with the lowest energy results in the early death of such nodes, which leads to significant packet drop as the network operates. The improved IA-IEEDBR (iIA-IEEDBR) protocol addresses the early death of low-energy nodes in the IA-IEEDBR protocol [25]. It divides the network into four logical sections with each section having a header that controls the death of nodes. All nodes are randomly deployed in all sections. However, the number of nodes in a single section is divided into two equal numbers of sleeping and sensing nodes. When a node dies in a logical section, the header node turns a sleeping node into a sensing node to avoid data loss. However, the header nodes are overloaded, and their death collapses the performance of the entire network. Furthermore, transforming a sleeping node into a sensing node when a node dies in a section does not guarantee the uninterrupted forwarding of data. This is because a node may die at a critical position and another node may be transformed into a sensing node in a less critical  position.

This paper contributes in a number of ways. The relay selection is accomplished on the basis of the pressure level (depth) and response time. A sender node chooses a neighbor node as a relay node with the lowest pressure level. If two or more neighbor nodes have the same pressure levels, the response time is taken into consideration. In this case, a neighbor node with the lowest pressure level and shortest response time is considered as a relay node. Unlike the conventional protocols such as DBR and EHRDBR, this strategy ensures that data packets follow the path with the least latency from the source to the destination. At the same time, it does not require the full-dimensional localization information of sensor nodes.

Instead of finding the position of energy holes, which is challenging and inefficient in underwater communications, a sender node increases its transmission range. This increase in the transmission range is accomplished when a sender node does not find any one-hop neighbor or when all its one-hop neighbors are dead. A unique packet holding time is defined for every node on the basis of the pressure difference between the sender and receiver, the ratio of the current and consumed energy, and the number of neighbors of the receiver node. This holding time ensures that nodes close to the surface of the water with a low current energy and lower number of neighbors hold the packet for short time. This, in consequence, leads to less interference and successful packet delivery to the final destination.

## 3. Channel Model

### 3.1. Channel Noise

Noise in underwater communications is constituted by four components: shipping, wave, thermal and turbulence noise [26]. The power spectral density (PSD) of each component in decibels is denoted by $N_{tab}$, $N_{sh}$, $N_{wv}$ and $N_{th}$, respectively. The PSD of the total noise is the sum of the individual PSD of each noise component and is given by

(1) $$N=N_{sh}+N_{wv}+N_{th}+N_{tb}$$

The individual PSD of each noise component is modeled by

(2) $$N_{sh}=40+20\left(s-0.5\right)+26logf-60log\left(f+0.03\right)$$

(3) $$N_{wv}=50+{7.5w}^{0.5}+20logf-40log\left(f+0.4\right)$$

(4) $$N_{th}=-25+20logf$$

(5) $$N_{tb}=27-30logf$$

The unit of frequency *f* is kilohertz. The dimensionless variable *s* is called the shipping parameter and has values in the range [0,1]. It measures the shipping activities on the surface of water. These shipping activities generate shipping noise in the range of 10–100 Hz. When wind blows at the surface of water with a speed *w* in meters per second, the resulting generated noise in the range from 100  Hz to 100 kHz is called wave noise. Most of the acoustic communications are affected by wave noise. Thermal noise dominates above 200 kHz. Turbulence noise in the 0.1–10 Hz range is the result of turbulence in sea water.

### 3.2. Channel Attenuation

The underwater medium reduces the strength of the desired signal when it travels away from the source. This reduction in strength, also called path loss, is modeled by attenuation. The attenuation A(d,f) of a signal of frequency *f* at a distance *d* from the source is modeled by the following [27]:

(6) $$A\left(d,f\right)=A_{0}d^{k}{\alpha (f)}^{k}$$

where $A_{0}$, *k* and α are the unity normalization constant, spreading factor and the absorption coefficient, respectively. As a result of variations in absorption, attenuation is often conveniently expressed in decibels as

(7) $$10logA\left(d,f\right)/A_{0}=k\cdot 10logd+d\cdot 10log\alpha (f)$$

For significantly higher frequencies in kilohertz, Thorp empirically models the absorption coefficient in decibels per kilometer as

(8) $$10log\alpha (f)=0.11\frac{f^{2}}{1+f^{2}}+44\frac{f^{2}}{4100+f^{2}}+2.75\cdot 10^{-4}f^{2}+0.003$$

and for smaller frequencies as

(9) $$10log\alpha (f)=0.002+0.11\frac{f^{2}}{1+f^{2}}+0.011f^{2}$$

### 3.3. The Speed of Acoustic Waves

The underwater channel is highly unpredictable and possesses properties that affect the speed of acoustic waves during underwater communications. The speed of acoustic waves varies in response to the characteristics of sea water as the medium. The salinity *S* in ppt (parts per thousand), temperature *T* in ${}^{\circ }$C and depth *D* in meters all affect the speed of acoustic waves in sea water. Mathematically, it is modeled by the following [28]:

(10) $$\begin{array}{ccc} c & = & 1449+4.591T-5.304\times 10^{-2}T^{2}\\ & & +2.374\times 10^{-4}T^{3}+1.34\left(S-35\right)\\ & & +1.63\times 10^{-2}D+1.675\times 10^{-7}D\\ & & +1.025\times 10^{-2}T\left(S-35\right)-7.139\times 10^{-3}TD^{3} \end{array}$$

where *c* is the speed of acoustic waves in water and the other parameters have the same description as above. Contrary to the higher speed of radio waves, the slower speed of acoustic waves results in inherent delay in underwater communications. The temperature, salinity and depth limitations for the above equation to be applicable are 0 ${}^{\circ }$C $<T\leq 30{\;}^{\circ }$C, 30–40 ppt and 0 m ≤D≤ 8000 m, respectively. The proposed scheme meets all of these conditions while performing routing. The proposed network has a depth of 1000 m that leads to variations in the speed of acoustic waves during protocol operation. Consequently, the above relation is used to measure the speed of acoustic waves during data transmission and delay measurement.

### 3.4. Bandwidth

The use of acoustic waves reduces the available bandwidth in underwater communications. This is because the acoustic bandwidth is limited and most of the frequencies are severely attenuated by water. Consequently, increasing the bandwidth reduces the effectiveness of underwater networks, as shown in Table 1 [7].

The network convergence and range decrease as the bandwidth increases. As a result, underwater networks with a high bandwidth do not cover a significant part of the sea to deploy and are, therefore, not effective in covering the sea environment. As a result of the absorption of the radio waves in water, the acoustic waves used in underwater communications have a limited spectrum. This spectrum is also severely affected by the properties of the underwater medium. Consequently, the underwater channel is associated with low data rates. As shown in Table 1, the proposed protocol operates in the short convergence range, as the network has a depth of 1000 m.

## 4. Proposed Protocol

### 4.1. The Proposed Network Model

**Figure 1:**
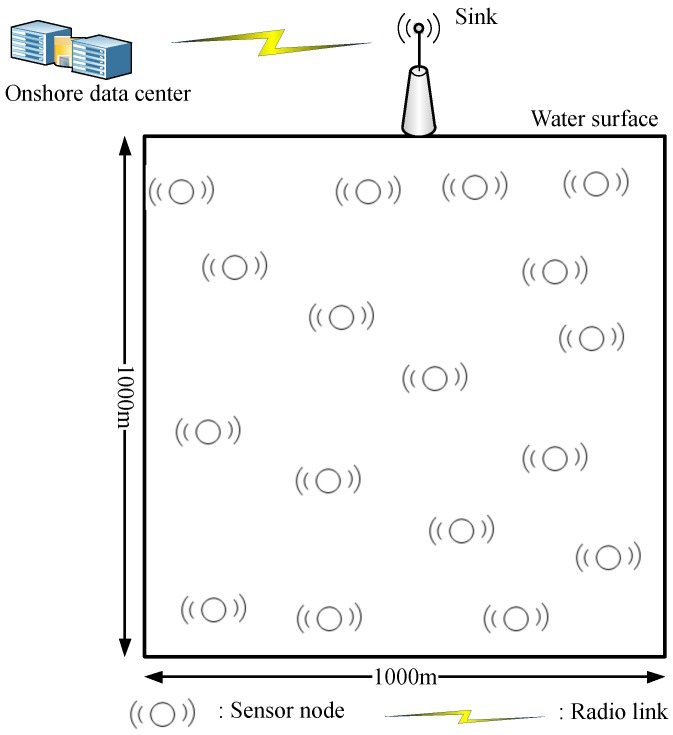
Network model.

The proposed network randomly deploys sensor nodes in a cube with a face length of 1000 m. The sink resides at the top middle surface of the network, as shown in Figure 1.

Nodes are capable of communicating with each other through acoustic waves, as radio waves are more poorly affected by water. The sink uses both acoustic and radio waves. It communicates with sensor nodes through the acoustic waves and with the onshore center through the radio waves. On account of a higher speed of the latter, data that the sink receives is considered as delivered to the onshore data center.

### 4.2. Neighbor Determination

**Figure 2:**
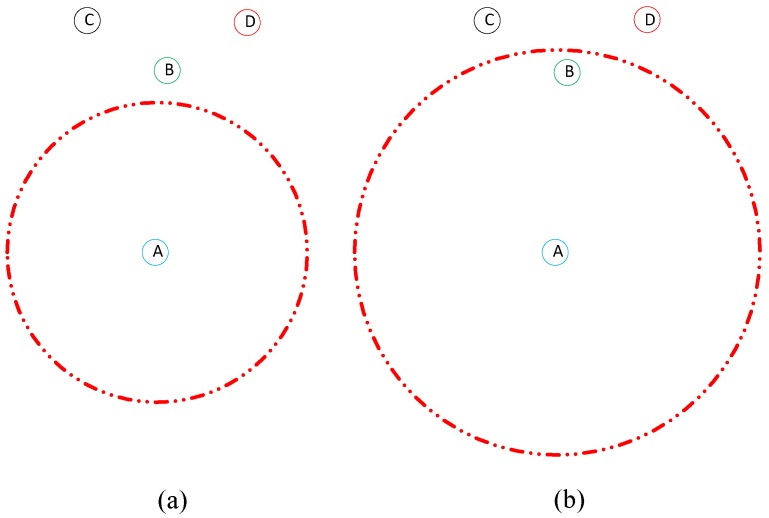
Adaptive neighbor determination. (**a**) Node A has no neighbor within its transmission range. (**b**) Node A increases its transmission range to include one or more neighbors.

After the random deployment of sensor nodes, they exchange hello packets. Initially just after deployment, nodes do not know their neighbors. In order to identify its neighbors, every sensor node broadcasts a hello packet that contains its measured pressure level and unique ID. It waits for a certain time to hear from its neighbors: nodes that are within its transmission range. This waiting time is modeled by

(11) $$\tau_{w}=\tau_{p}+\tau_{pr}$$

where $\tau_{w}$, $\tau_{p}$ and $\tau_{pr}$ represent the waiting time, propagation delay and processing delay, respectively. The propagation delay depends upon the distance between the transmitter and receiver and the speed of the acoustic wave. It is modeled by

(12) $$\tau_{p}=\tau_{sr,i}+\sum_{i,j\in n}\tau_{i,j}+\tau_{j,snk}\;\;n\geq 2,\;i,j\in n$$

(13) $$\tau_{p}=\tau_{sr,i}+\tau_{i,snk}\;\;n=1,\;i\in n$$

(14) $$\tau_{p}=\tau_{sr,snk}\;\;n=0$$

where *n* is the number of hops for a packet and $\tau_{sr,i}$, $\tau_{i,j}$, $\tau_{j,snk}$ and $\tau_{sr,snk}$ are the propagation delays from a sender node to a forwarder *i*, forwarder *i* to forwarder *j*, forwarder *j* to the sink and from the sender to the sink, respectively. Upon receiving a response from a neighbor, the broadcaster node initially obtains the information about the neighbors’ pressure levels and IDs. It then constructs a table that contains its number of neighbors with their corresponding IDs and pressure levels. The table is broadcasted. Every node undergoes this process. In this way, not only does a sensor node know about its own number of neighbors but its one-hop neighbors also know about it. When a broadcaster node does not hear back from any node within the waiting time, it sends the hello packet again and waits to receive a response from its neighbors. If it does not receive any response from its neighbors within the maximum waiting time $max(t_{w})$, it adaptively increases its transmission range to the maximum threshold and rebroadcasts the hello packet to include one or more neighbors, as shown in Figure 2. In case (a), a sender node A has data to send but there is no node within its transmission range. Nodes B, C and D are in the proximity of its transmission range but not within it. Knowing this, node A increases its transmission range until node B lies within its range (becomes its neighbor), as shown in case (b).

The transmission range is always increased to the maximum threshold independently of how many new nodes lie in it. Node A (and every other sensor node) declares no neighbor at all when the maximum number of rebroadcasts are reached with no neighbor at all even after increasing the transmission range. The neighbor-finding process is repeated after regular intervals of time as nodes die due to their limited battery life and change their positions with water currents. This ensures that data is forwarded to live neighbors, which reduces the probability of packet loss.

### 4.3. Packet Forwarding

When a sensor node has a data packet to send, it chooses the best forwarder among its neighbors by looking into the routing table. The best forwarder has the lowest pressure level. The sender node inserts the ID of the best forwarder into the data packet and forwards it. All its neighbors receive it. Every neighbor matches its own ID with the ID of the best forwarder in the data packet. The intended forwarder node accepts the packet for further transmission towards the sink. All the rest of the neighbors simply discard it because of a mismatch of the IDs. This process continues until the packet reaches the surface sink or drops if the link is not free within the maximum back-off time. If a sender node has two or more forwarder nodes with the same pressure levels, the response time is taken into account to select the best forwarder. In such a case, the best forwarder has the lowest pressure level and the shortest response time.

During packet forwarding, the propagation delays defined by Equations (12)–(14) are calculated for multi-hop, one-hop and direct (from the sender to the sink) communications, respectively. For multi-hop communications, *n* should be greater than or equal to 2, as at least two forwarders should forward a packet. In the calculation of the response time, the direct communications from the broadcaster node to its neighbors are considered. The processing delay is an inherent parameter of the sensor design. It is a measure of the time difference between the reception of a hello packet to the initiation of the response to the original broadcaster. Possessing the same characteristics, it is assumed that the processing delay is the same for all nodes.

Algorithm 1 shows the selection of the best relay. A sender node *i* chooses the best relay node *j* among its set of neighbors $N_{i}$ with the lowest pressure level $min(p_{j})$ or with the lowest pressure level and shortest response time $min(p_{j},\tau_{w})$.

**Algorithm 1** The Best Relay SelectionBR←thebestrelay$p_{j}\leftarrow \mathrm{pressure}\;\mathrm{level}\;\mathrm{of}\;a\;\mathrm{forwarder}\;\mathrm{node}\;j$R←transmissionrangeofasensornodeE←energyofasensornode$N_{i}\leftarrow \mathrm{number}\;\mathrm{of}\;\mathrm{neighbors}\;\mathrm{of}\;a\;\mathrm{source}\;\mathrm{node}\;i$$N_{j}\leftarrow \mathrm{number}\;\mathrm{of}\;\mathrm{neighbors}\;\mathrm{of}\;a\;\mathrm{relay}\;\mathrm{node}\;j$M←totalnumberofnodesinthenetwork**for** i=1:1:M **do**  **if** $E_{i}>0\;\;\;\&\;\;\;E_{j}>0\;\;\;\&\;\;\;j\in N_{i}$ **then**   $BR_{i}=argmin_{j\in N_{i}}\left(p_{j}\right)\;\;\;OR\;\;\;argmin_{j\in N_{i}}\left(p_{j},\tau_{w}\right)$  **else**all nodes are dead  **end if****end for**

### 4.4. Packet Holding Time

Upon the reception of a data packet by an intended suitable forwarder *j* from a sender node *i*, the node holds it for a certain duration of time called the packet holding time $\tau_{h}$. This time depends upon the number of neighbors $N_{j}$ of the forwarder, the pressure difference between the sender and forwarder $p_{i}-p_{j}$, the speed of the acoustic wave *c* and the ratio of the initial energy level $E_{0}$ to the current energy level $E_{c}$. Mathematically, it is written as

(15) $$\tau_{h}=\frac{N_{j}\left(p_{i}-p_{j}\right)}{c(\frac{E_{0}}{E_{c}})}$$

The packet holding time modeled above ensures that a packet is reached from a source to a destination with a small delay, a low interference and a low probability of loss. Its dependency on the pressure difference ensures that low-depth nodes close to the surface of the water hold packets for shorter time, as these nodes are often overburdened by the nodes in the bottom. This increases the probability of successful packet transmission towards the sink by reducing the overloading of these nodes by data packets. If such nodes hold the packets for a long period of time, this results in overloading and congestion that finally will result in packet loss. Additionally, a forwarder node having a lower number of neighbors will hold the packet for a shorter time than a node with a greater number of neighbors. This is because the former faces less interference (less neighbors) than the latter (more neighbors). Furthermore, the ratio $\frac{E_{0}}{E_{c}}$ is smaller for a node with a higher current energy level than a node with a smaller value of its current energy level. Therefore, nodes with greater values of current energy levels hold the packets for a longer time, as these nodes have enough energy to remain alive in the network. A node holds a packet and senses the channel to become free to transmit the packet.

**Figure 3:**
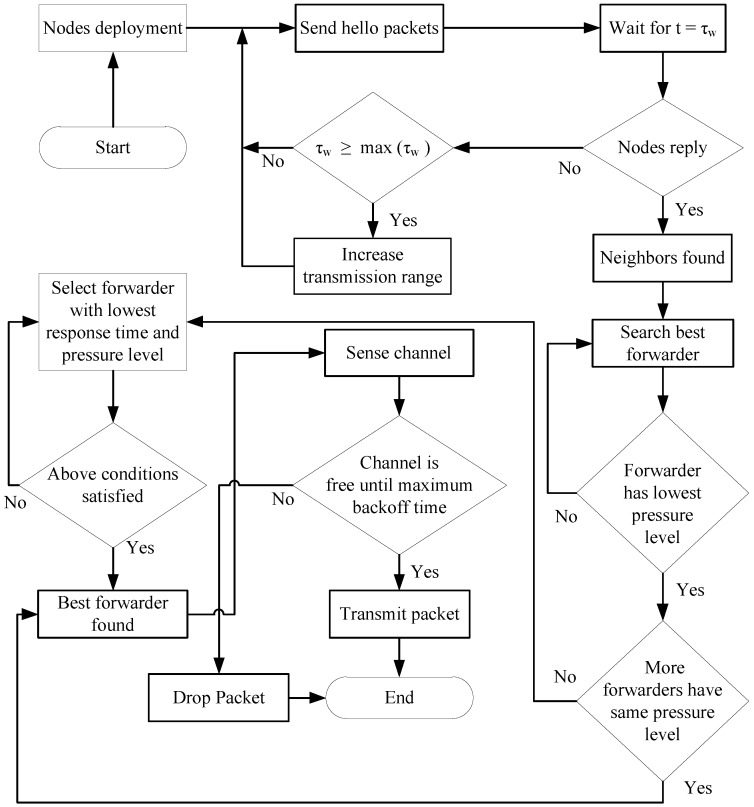
Flow chart of the proposed scheme.

If the channel is not free, the node backs off. The packet is ultimately dropped when the maximum back-off time is reached. Figure 3 shows the flow chart of the proposed scheme as described above.

## 5. Simulation Results

The simulation was performed using MATLAB. The proposed network is a cube of dimensions 1000 m × 1000 m × 1000 m in which 200 nodes are randomly deployed. Underwater communications usually deploy few source nodes at the bottom of the network, as in DBR and EEDBR [29]. Therefore, the proposed scheme considers two source nodes at the bottom of the network. These nodes sense the desired attribute and convert it into packets. Packets are generated at the rate of one packet per second. The size of a single packet is 50 bytes. Among the available modems [30], every node uses the LinkQuestUWM2000 modem to communicate with other nodes [31] and has an initial energy of 70 J [29]. This modem has a data rate of 10 kbps and consumes 8 W, 0.8 W and 8 mW power in transmit, receive and idle mode, respectively. Additionally, it can work up to a maximum depth of 2000 m (or 4000 m). The beam width in the omni-directional mode of the transducer is $210^{\circ }$. This eases the selection of a forwarder node close to the surface of the water for a sender node. On the basis of the specifications of the modem, the transmission range of a node is 300 m in all directions and can be increased to 450 m to avoid the condition in which no neighbor node is found. These features of the modem make it the best choice for the proposed protocol. For each measurement, 20 simulations were performed. The NEFP and EHRDBR protocols were chosen for comparison with the proposed protocol because of their similarity to the proposed protocol. The NEFP protocol uses the forwarding probability of a packet and the packet holding time to route data packets in a restricted forwarding zone. This zone is defined by the angle formed among the source, relay and destination. The proposed protocol also uses the packet holding time to minimize interference. However, the proposed protocol defines the holding time in a unique manner. The EHRDBR first detects a dead node (energy hole) and then moves an active node to replace the dead node. The proposed protocol, in contrast, increases the transmission range to include one or more active nodes in the transmission range of a sender node. Following the DBR protocol, the random walk mobility model is considered to take into account the movements of sensor nodes with water currents. According to this model, nodes move in random directions with a speed that ranges from 1 to 5 m/s. For the MAC (medium access control)layer, the 802.11-DYNAV protocol is considered [32]. The performance metrics are described below.

- **Round:** The time that lapses from the transmission of a single or more packets by one or more source nodes to its successful reception at the sink or drop.
- **Total Energy Consumption:** This is the amount of energy consumed by all live nodes in one round. It includes energy consumption during hello packets’ exchange, transmission and reception of a packet and while remaining in the idle state.
- **Dead Nodes:** Sensor nodes that consume all the initially assigned energy.
- **Live Nodes:** Sensor nodes that have not yet consumed all the initially assigned energy.
- **End-To-End Delay:** This is the time taken by a data packet from transmission by a source node to reception at destination.
- **Packet Delivery Ratio:** Ratio of the total packets received successfully at the sink to the total packets transmitted.

**Figure 4:**
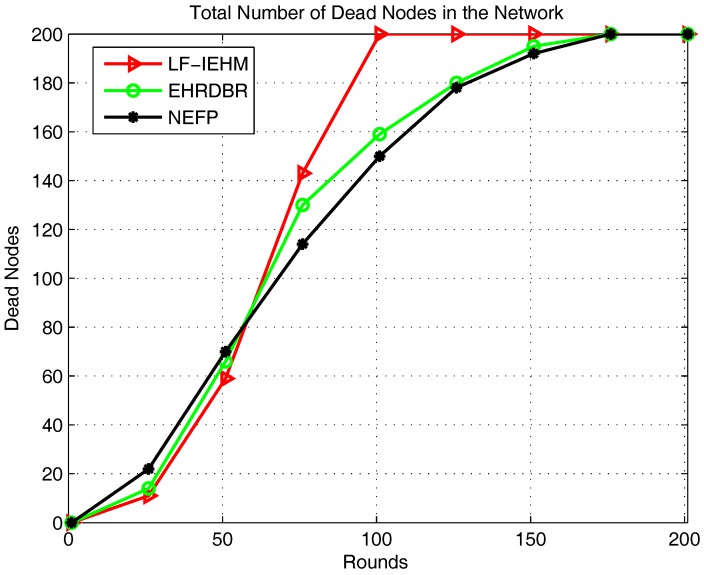
Total number of dead nodes in the network.

Figure 4 shows the plot of the total number of dead nodes in the network. For almost the first 58 rounds, nodes died with the slowest rate in the LF-IEHM. This is due to the availability of more live forwarder nodes. This allows a sender node to select forwarder nodes within its normal transmission range, consuming less energy. In addition, the use of the response time (in addition to the pressure levels of the forwarder nodes when multiple forwarder nodes have the same pressure levels) ensures that a single forwarder node is not constantly selected by a sender node in the proposed protocol. This behavior avoids the overburdening of the same forwarder node repeatedly that, in turn, results in its rapid death. The response time of a forwarder changes when it moves with water currents. This, in consequence, ensures that multiple nodes are selected as forwarders and a single node is not overburdened and made depleted of its battery power. This behavior is not addressed in the NEFP or EHRDBR protocols. As a result, nodes died with the slowest rate in the LF-IEHM protocol for almost the first 58 rounds. As the rounds progressed, nodes began dying, particularly nodes close to the water surface with a low pressure level. This causes the formation of energy holes, which results in packet drop. In order to overcome these energy holes, a sender node in LF-IEHM increases its transmission range. This results in a greater energy consumption by nodes. Consequently, nodes died with the highest rate in LF-IEHM after 58 rounds. Nodes died with the highest rate in the NEFP for almost the first 58 rounds. This was because the NEFP selects forwarders close to the water surface (the sink) and within a restricted zone. Such nodes are selected frequently for data forwarding, which makes them depleted of their energy and causes them to die. After 58 rounds, nodes died at a faster rate in EHRDBR than in the NEFP because of the death of overburdened low-depth nodes in EHRDBR and the extra cost of energy hole repairing.

**Figure 5:**
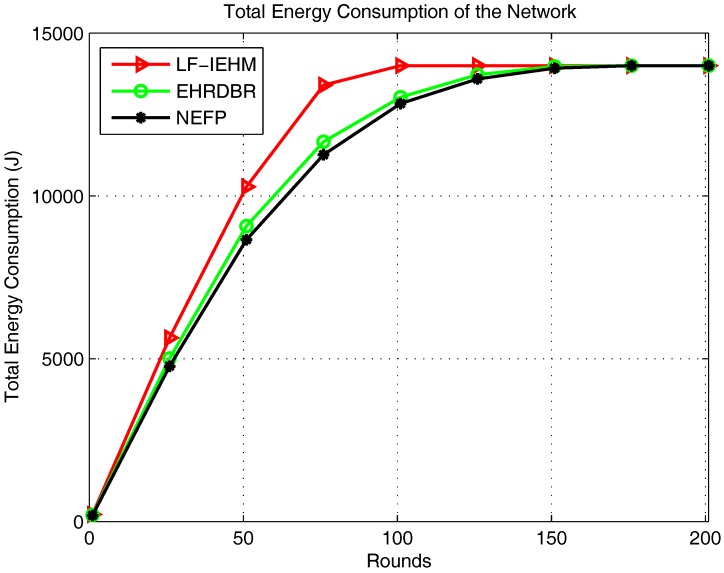
Total energy consumption of the network.

**Figure 6:**
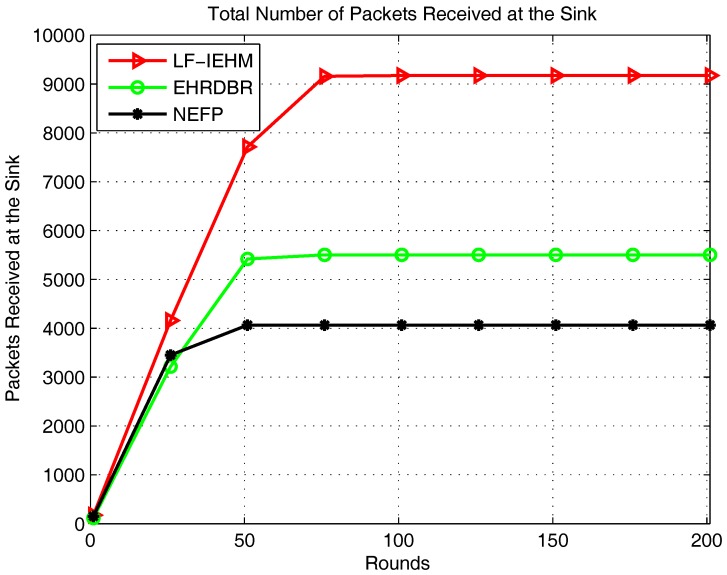
Total number of packets received at the sink.

Figure 5 shows the plot of energy consumption. Initially, for the first several rounds, the energy consumption was almost the same in all of the protocols. This was because more forwarder nodes were available to route data along the best available paths in all the protocols. After this, the nodes began to die, and the best available paths were no longer available. Following this, a sender node in LF-IEHM increased its transmission range and consumed more power to select one or more forwarder nodes for data forwarding. This increase in the transmission range takes place when a sender node does not find any neighbor node to select it as a forwarder node. This process avoids packet loss due to unavailability of forwarder nodes at the expense of more energy consumption. This leads to the highest energy consumption in LF-IEHM. The energy consumption curve of LF-IEHM shows remarkable deviation from the corresponding curves of the EHRDBR and NEFP. protocols between the 22nd and 100th rounds. This means LF-IEHM consumes energy with the fastest rate in this interval of rounds. This is because sender nodes begin to consume more power by increasing their transmission range to forward packets as forwarder nodes die in their neighborhood. The sender nodes in the NEFP select forwarder nodes in a restricted zone. In contrast, the sender nodes in EHRDBR select forwarder nodes in the full transmission range. Additionally, EHRDBR involves redundant packet transmission. The hole repairing also consumes energy in EHRDBR. As a result, the energy consumption is higher in EHRDBR than in the NEFP. Figure 6 shows the total number of packets received at the sink. As a result of the variable transmission range and unique packet holding time of the nodes, LF-IEHM had the greatest number of packets received at the sink. In LF-IEHM, when a sender node does not find any neighbor, it increases its transmission range to include one or more forwarder nodes. This increases the probability of the packets’ reception at the sink. It also ensures the minimization of packet loss when the nodes close to the water surface die. In addition, the unique packet holding time of every node ensures that no two or more nodes forward data packets at the same time. This reduces interference during data forwarding, which, in turn, minimizes packet loss. As a result, LF-IEHM had the largest number of packets received at the sink compared to EHRDBR and the NEFP, for which every node has a fixed transmission range. Initially for almost the first 25 rounds, the number of packets received at the sink was roughly the same for EHRDBR and the NEFP. This was due to the availability of the best forwarder nodes in both protocols. As the rounds progressed, nodes began to die in both protocols. However, the energy hole repairing process replaces the dead nodes by active nodes in EHRDBR. This reduces the packet loss in EHRDBR. As a result, the number of packets received at the sink became higher in EHRDBR than in the NEFP.

**Figure 7:**
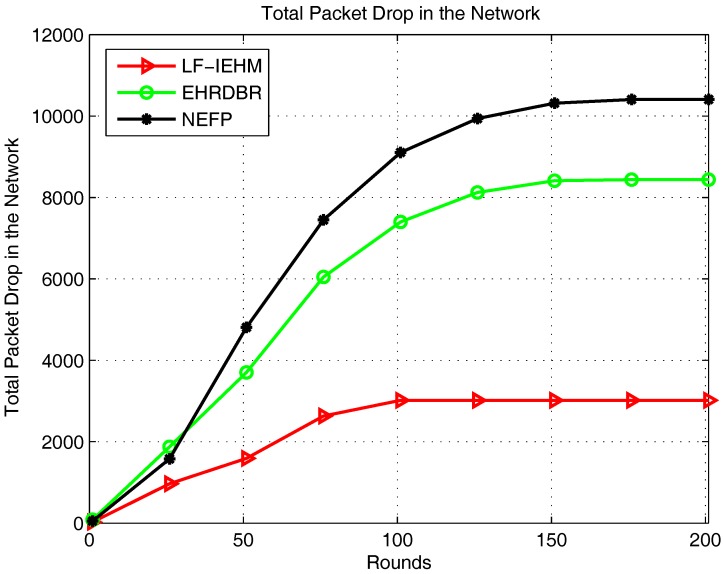
Total packet drop in the network.

**Figure 8:**
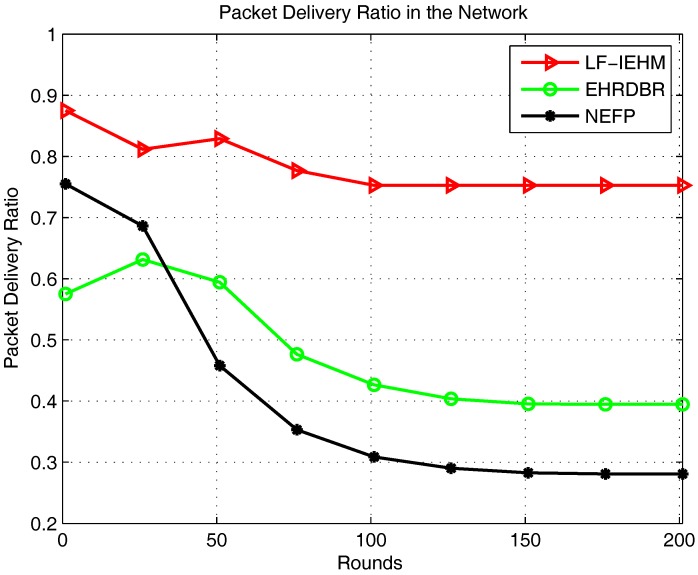
Packet delivery ratio in the network.

**Figure 9:**
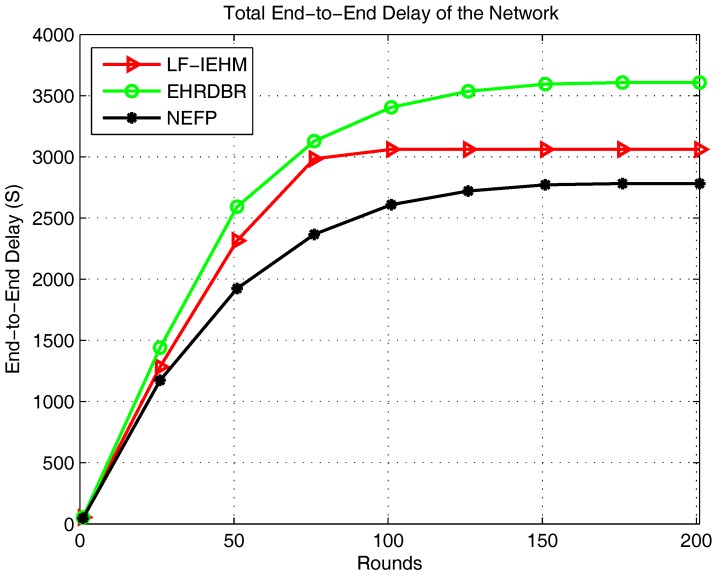
Total end-to-end delay of the network.

Figure 7 shows the plot of the total packet drop. As a result of a variable transmission range and interference mitigation strategy, LF-IEHM had the lowest packet drop. This was because when a sender node does not find any neighbor node to forward data packets to, it increases its transmission range to include one or more neighbors. This reduces the packet drop. Furthermore, the unique holding time of every node minimizes the simultaneous transmission of packets by two or more nodes. This results in packet drop due to interference. Initially, the NEFP had a lower packet drop than EHRDBR because of forwarding packets in the restricted zone on the basis of the forwarding probability. This approach forwards the packets from the source to the destination along the shortest paths and avoids packet routing over unnecessary paths. This, in turn, reduces the probability of packet drop. However, forwarder nodes soon die in the restricted zone in the NEFP because of the frequent forwarding of data. At the same time, when nodes begin dying in EHRDBR, its energy hole repairing mechanism replaces energy holes with live nodes. Additionally, redundant packet transmission in EHRDBR reduces the probability of packet drop. In essence, packet drop decreased in EHRDBR and increased in the NEFP in later rounds. Figure 8 shows the plot of the packet delivery ratio. Following the pattern of packets received at the sink and packet drop, LF-IEHM had the highest packet delivery ratio. The delivery ratio was higher for the NEFP than EHRDBR for the first several rounds but decreased as the rounds progressed as a result of the death of nodes in the restricted forwarding zone in the NEFP and energy hole repairing and redundant packet transmissions in EHRDBR. Figure 9 shows the plot of end-to-end delay. Due to forwarding in the restricted forwarding zone from source to destination, NEFP has the smallest end-to-end delay. In EHRDBR, a sender node selects forwarder nodes within the depth threshold. This caused the greatest end-to-end delay in EHRDBR. LF-IEHM had a greater delay than the NEFP as a result of the selection of forwarder nodes in the full transmission range rather than in a restricted zone as in the NEFP. The selection of forwarder nodes in the restricted zone results in choosing the shortest possible paths from a source to a destination. This reduces the end-to-end delay. LF-IEHM had a shorter end-to-end delay than EHRDBR because the former takes into account the response time of the nodes in addition to the depth (water pressure level) as in EHRDBR. This ensures that when two or more forwarder nodes have the same depth, the sender node chooses the forwarder node with the shorter response time. As a result, the end-to-end delay was smaller in LF-IEHM than in EHRDBR.

## 6. Conclusions and Future Work

A LF-IEHM protocol has been designed for UWSNs to address energy hole formation and interference that threaten the reliable delivery of packets from a source to a destination. An increase in the transmission range of a sensor node overcomes the creation of energy holes. The uniquely defined packet holding time for every sensor node reduces the simultaneous transmission of packets by two or more forwarders. This, in turn, reduces the interference. Simulation results have revealed the superior performance of the proposed scheme in terms of the addressed issues. As future work, a cross-layer design for interference mitigation can be considered by making the MAC layer more intelligent to cope with interference. In addition, mobile sinks can be used or nodes close to the water surface can be powered using energy harvesting, as these nodes die early as a result of heavy data traffic.

## Figures and Tables

**Table 1:** Bandwith and transmission range relationship for underwater wireless sensor networks (UWSNs).

Convergence	Range (km)	Bandwidth (kHz)
Very long	100	Less than 1
Long	10–100	2–5
Medium	1–10	Almost 10
Short	0.1–1	20–50
Very short	Less than 0.1	Greater than 100

## References

[1] Pompili D., Tommaso M. Three-dimensional routing in underwater acoustic sensor networks. Proceedings of the ACM 2nd International Workshop on Performance Evaluation of Wireless Ad Hoc, Sensor, and Ubiquitous Networks.

[2] Shojafar M., Cordeschi N., Baccrelli E. (2016). Energy-efficient adaptive resource management for real-time vehicular cloud srvices. IEEE Transac. Cloud Comput..

[3] Naranjo P.G.V., Shojafar M., Mostafaei H., Pooranian Z., Baccarelli E. (2017). P-SEP: A prolong stable election routing algorithm for energy-limited heterogeneous fog supported wireless sensor networks. J. Supercomput..

[4] Ahmadi A., Hajeforosh M.F., Dehghan M., Singhal M. (2013). An efficient routing algorithm to preserve k-coverage in wireless sensor networks. J. Supercomput..

[5] Anisi M.H., Abdullah A.H. (2016). Efficient data reporting in intelligent transportation systems. Netw. Spat. Econ..

[6] Heidemann J., Ye W., Wills J., Syed A., Li Y. Research challenges and applications for underwater sensor networking. Proceedings of the IEEE Wireless Communications and Networking Conference.

[7] Akyildiz I.F., Pompili D., Melodia T. (2005). Underwater acoustic sensor networks: Research challenges. Ad Hoc Netw..

[8] Yang X. (2010). Underwater Acoustic Sensor Networks.

[9] Latif K., Javaid N., Ahmad A., Khan Z.A., Alrajeh N., Khan M.I. (2016). On energy hole and coverage hole avoidance in underwater wireless sensor networks. IEEE Sens. J..

[10] Latif K., Saqib M.N., Bouk S.H., Javaid N. Energy hole minimization technique for energy efficient routing in under water sensor networks. Proceedings of the Springer International Conference on Communication Technologies, Information Security and Sustainable Development.

[11] Khan A., Javid N., Mahmood H., Khan S., Khan Z., Qasim A. EEIRA: An energy efficient interference and route aware protocol for underwater WSNs. Proceedings of the IEEE 10th International Conference on Complex, Intelligent, and Software Intensive Systems.

[12] Shashaj A., Petroccia R., Petrioli C. Energy efficient interference-aware routing and scheduling in underwater sensor networks. Proceedings of the IEEE Oceans.

[13] Heidemann J., Stojanovic M., Zorzi M. (2011). Underwater sensor networks: Applications, advances and challenges. Philos. Trans. R. Soc. A.

[14] Urick R.J. (1967). Principles of Underwater Sound for Engineers.

[15] Garcia M., Sendra S., Atenas M., Lloret J., Loo J., Mauri J.L., Ortiz J.H. (2011). Underwater wireless ad-hoc networks: A survey. Mobile Ad Hoc Networks: Current Status and Future Trends.

[16] Qingwen W., Fei C., Zhi L., Qian Q. A novel efficient forwarding protocol for 3-D underwater wireless sensor networks. Proceedings of the IEEE 11th International Conference on Industrial Electronics and Applications.

[17] Majid A., Azam I., Khan T., Sangeen, Khan Z.A., Qasim U., Javaid N. A reliable and interference-aware routing protocol for underwater wireless sensor networks. Proceedings of the IEEE 10th International Conference on Complex, Intelligent and Software Intensive Systems.

[18] Fahim H., Javaid N., Qasim U., Khan Z.A., Javed S., Hayat A., Iqbal Z., Rehman G. Interference and bandwidth aware depth based routing protocols in underwater WSNs. Proceedings of the IEEE 9th International Conference on Innovative Mobile and Internet Services in Ubiquitous Computing.

[19] Yan H., Shi Z., Cui J. DBR: Depth-based routing for underwater sensor networks. Proceedings of the 7th International IFIP-TC6 Networking Conference on Ad Hoc and Sensor Networks, Wireless Networks, Next Generation Internet.

[20] Li D., Du J., Liu L. A data routing algorithm based on Markov model in underwater wireless sensor networks. Proceedings of the IEEE 16th International Conference on Ubiquitous Wireless Broadband.

[21] Ghoreyshi S.M., Shahrabi A., Boutaleb T. An opportunistic void avoidance routing protocol for underwater sensor networks. Proceedings of the IEEE 30th International Conference on Advanced Information Networking and Applications.

[22] Basagni S., Petrioli C., Petroccia R., Spaccini D. (2015). Carp: A channel-aware routing protocol for underwater acoustic wireless networks. Ad Hoc Netw..

[23] Noh Y., Lee U., Wang P., Vieira L.F.M., Cui J.-H., Gerla M., Kim K. (2016). Hydrocast: Pressure routing for underwater sensor networks. IEEE Trans. Veh. Technol..

[24] Shah M., Javaid N., Imran M., Guizani M., Khan Z.A., Qasim U. Interference aware inverse EEDBR protocol for underwater WSNs. Proceedings of the 11th International Conference on Wireless Communications and Mobile Computing Broadband.

[25] Shakeel U., Javaid N., Ejaz M., Zarar S., Hafeez T., Muhammad Improved interference-aware EEDBR protocol for underwater wireless sensor networks. Proceedings of the IEEE 10th International Conference on Broadband and Wireless Computing, Communication and Applications.

[26] Etter P. (2013). Underwater Acoustic Modeling and Simulation.

[27] Coates W.R.F. (1989). Underwater Acoustic Systems.

[28] Mackenzie K.V. (1981). Nineterm equation for sound speed in the oceans. J. Acoust. Soc. Am..

[29] Wahid A., Lee S., Jeong H., Kim D. (2012). EEDBR: Energy-efficient depth-based routing protocol for underwater wireless sensor networks. Adv. Comput. Sci. Inf. Technol..

[30] Sendra S., Lloret L., Jimenez J.M., Parra L. (2016). Underwater acoustic modems. IEEE Sens. J..

[31] http://www.link-quest.com/html/uwm2000.htm.

[32] Shin D., Kim D. A dynamic NAV determination protocol in 802.11 based underwater networks. Proceedings of the IEEE 5th International Symposium on Wireless Communication Systems.

